# The Structure and Usage of Female and Male Mouse Ultrasonic Vocalizations Reveal only Minor Differences

**DOI:** 10.1371/journal.pone.0041133

**Published:** 2012-07-17

**Authors:** Kurt Hammerschmidt, Konstantin Radyushkin, Hannelore Ehrenreich, Julia Fischer

**Affiliations:** 1 Cognitive Ethology Laboratory, German Primate Center, Göttingen, Germany; 2 Division of Clinical Neuroscience, Max-Planck-Institute of Experimental Medicine, Göttingen, Germany; 3 Mouse Behavior Outcome Unit, Johannes Gutenberg University Mainz, Mainz, Germany; Utrecht University, Netherlands

## Abstract

Ultrasonic vocalizations (USV) of mice are increasingly recognized as informative dependent variables in studies using mouse models of human diseases. While pup vocalizations primarily serve to re-establish contact with the mother, adult male “songs” were considered to be courtship signals. Alternatively, mouse USVs may generally function as territorial signals. To distinguish between these two hypotheses, we compared the structure and usage of adult male and female USVs in staged resident-intruder encounters. If calls function primarily as courtship signals, males should respond stronger than females, specifically when presented with a female intruder. Refuting this hypothesis, we found that in response to female intruders, females called more than males (228±32 calls/min vs. 71±15 calls/min), and males called more to female than to male intruders (14±7.5 calls/min). There were no significant differences in the acoustic characteristics of the calls given by females and males. To control for the influence of the intruder's behavior on calling, we repeated the experiments using anaesthetized intruders. Again, females produced more calls to female than male intruders (173±17 calls/min vs. 71±15 calls/min), while males called more in response to female than male intruders (39±17 calls/min), and there were no acoustic differences in female and male calls. The vocal activity did not differ significantly with regard to intruder state (awake or anaesthetized), while the acoustic structure exhibited significant differences. Taken together, our findings support the view that calls do not mainly function as courtship signals, although they might serve both a territorial (sex-independent) and a courtship function. The comparison of responses to awake vs. anaesthetized intruders suggests that the latter are sufficient to elicit vocal activity. The subtle acoustic differences, however, indicate that the subject differentiates between intruder states.

## Introduction

In the last years, mouse ultrasonic vocalizations (USV) have received increasing attention, specifically as behavioral read-outs in genetic mouse models of human psychiatric disorders (e.g., [1]–[3]; reviewed in [4]), but also in studies of the genetic foundations of speech [5]. Studies targeted two general categories of calls: pup isolation calls and adult “songs”. Mouse pups emit isolation calls when removed from the mother, and in response to dropping body temperature, handling, or specific smells. In general, these calls are considered to be signals of need addressed to mothers [6]–[8]. Pup calls show some developmental modification, but auditory experience does not influence the structure of pup or adult calls later in life [9].

Fewer studies have examined the biological function of adult calls. Brudzynski [10] suggested that USVs evolved as an anti-predator adaptation that now serves to facilitate or inhibit social interaction [11], [12]. In contrast, Holy and Guo [13], [14] proposed that adult male songs function as courtship signals, but this interpretation has been questioned [15], [16]. The view that calls may serve a territorial function – that is to repel intruders or facilitate interaction and assessment – is supported by studies that examined female vocal behavior. Sales [17] reported that females emit USVs when paired with other females, a finding later replicated by Maggio and Whitney [18]. Recent studies using the resident-intruder paradigm, where a subject in its “home cage” is confronted with an intruding individual, showed that resident females emitted a comparable amount of USVs during these encounters as males [19]. In this design, the ‘resident’ animal was kept for one or more days in a ‘home’ cage. During the test, the ‘intruder’ was placed in the cage of the resident animal. The number of calls emitted by the resident seemed to be modulated by the motivational state of the caller. For instance, sexually receptive females emitted fewer ultrasonic vocalizations than non-receptive ones in the presence of a female intruder. In general, the results suggested that USVs emitted during such social interactions can be used as an indicator of social recognition. Scattoni and colleagues [1], [20] also found that USVs produced during resident-intruder tests could be used to characterize the social relationships between different females or to establish social dominance hierarchies [18].

In the present study, we aimed to shed more light on the function of adult mouse USVs by comparing both the usage and structure of calls given by males and females in resident-intruder encounters. If calls primarily serve as courtship signals, males should produce more calls than females when presented with a female intruder, and their calls should be more elaborate than females' calls. Moreover, males should produce more calls – and more elaborate ones – in response to female than male intruders. In case that calls mainly serve a territorial function or to establish social hierarchies, we would predict that females should respond in a similar way as males to female intruders. In terms of the acoustic structure, this hypothesis does not predict strong structural differences between the sexes. We measured the number of calls and latency to call; in addition, we characterized the call usage by performing a cluster analysis to identify call types and then calculated call type usage based on this objective classification of calls. Finally, we measured a suite of acoustic parameters to compare male and female vocalizations.

## Results and Discussion

### Experiment 1

**Figure 1:**
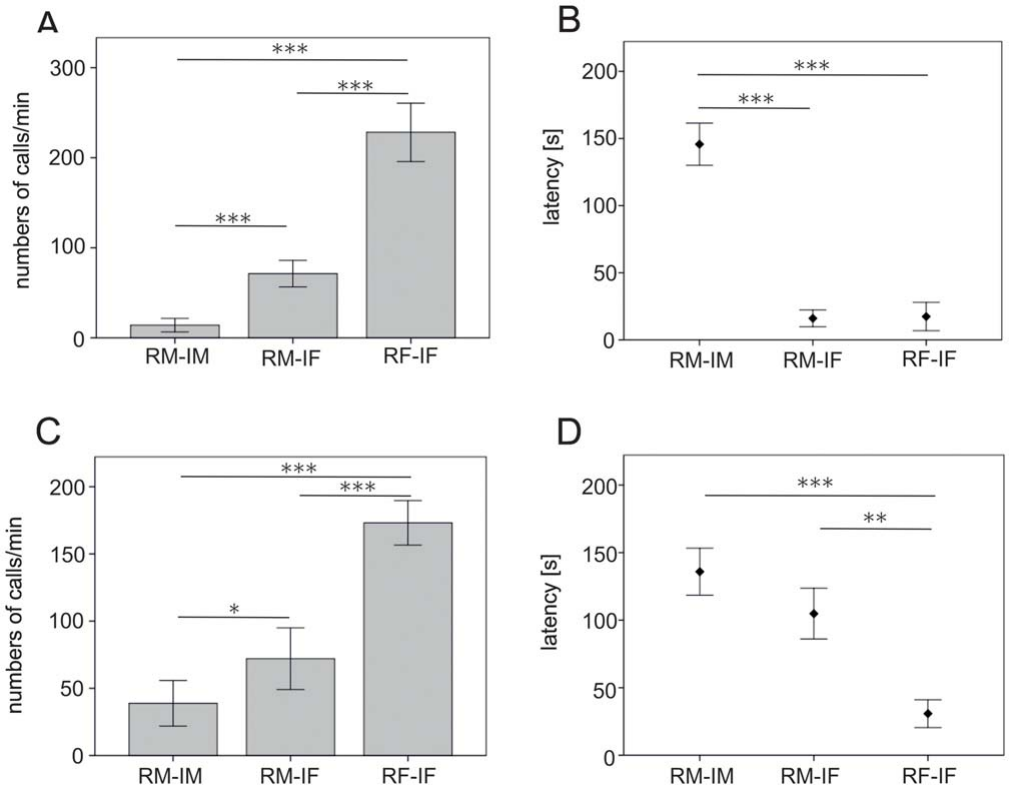
Number of calls and latency to call. A: Number of calls given in response to vivid intruder. B: Latency to call in response to vivid intruder. C: Number of calls given in response to anaesthetized intruder. D: Latency to call in response to anaesthetized intruder. Experimental conditions: RM = resident male, IM = intruder male, IF = intruder female, RF = resident female. Showa are mean and SEM. Significant differences: * <0.5, ** <0.01, < =  ***.

In response to female intruders, female mice called significantly more than males (t-test, t = −5.1, N = 28, P = <0.001), and overall produced the highest number of calls. Males called significantly more in response to female than male intruders (paired t-test: t = −4.5, N = 20, P = <0.001; Figure 1A). In response to female intruders, both females and males revealed a short latency until they began calling (t = −0.1, N = 24, P = 0.909), while the latency was significantly longer in males confronted with male intruders than with female intruders (t = 5.4, N = 20, P = <0.001; Figure 1B).

**Figure 2:**
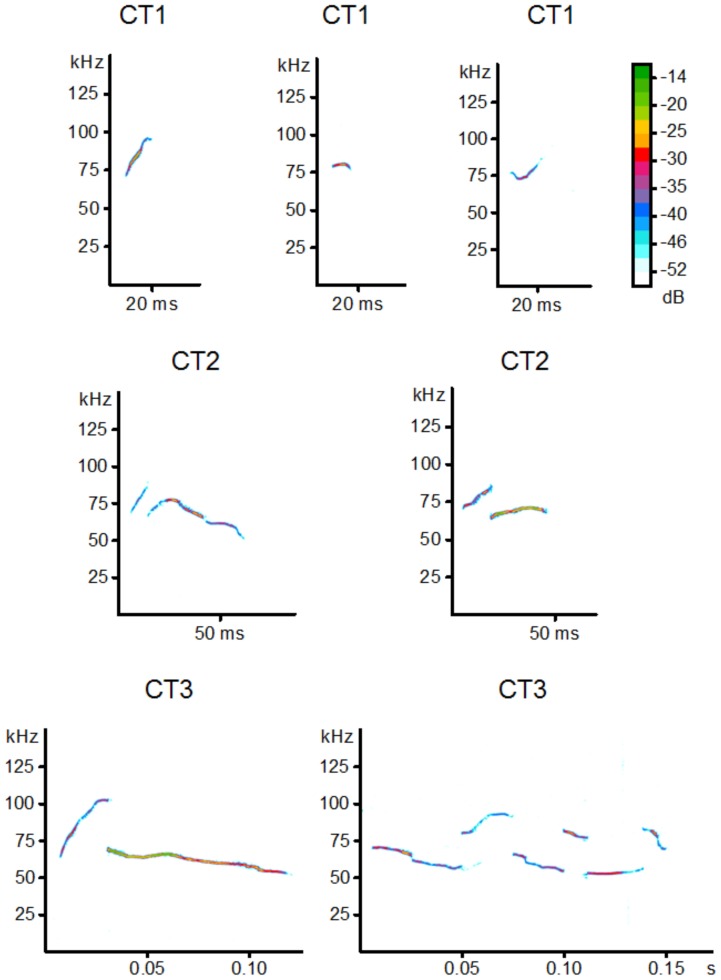
Spectrographic examples of different vocal types found by two-step cluster analysis (CA).

With the aid of a two-step cluster analysis, we identified three clusters corresponding to three different call types. The first cluster (CT1: 43.2% of calls) contained short calls (19.6±3.3 ms; mean ± SEM) mainly with increasing peak frequency and without major frequency jumps (9.2±0.25 kHz). The second cluster (CT2: 39.8%) consisted of calls of medium duration (40.1±0.7 ms), more or less decreasing peak frequency, and medium frequency jumps (14.8±0.54 kHz). The third cluster (CT3: 17%) contained calls with a long duration (95.3±1.6 ms) and high frequency jumps (28±0.54 kHz; Figure 2). A post-hoc discriminant function analysis correctly assigned 93.9% of the calls to the correct call category (cross validated: 93.9%, chance level: 33%). All three call types were used by females and males in all conditions (Table 1). However, males and females used calls from the CT1 category in significantly different proportions (Table 1). In terms of acoustic differences between male and female encounters, we found no significant differences in call duration, maximum peak frequency or any other of the tested variables (Table 2).

The results revealed that females responded more strongly than males and showed the same acoustic structure, refuting the assumption that these songs primarily serve as courtship signals. However, it might be the case that the calling behavior is also affected by the behavior of the intruder. Although an inspection of the spectrograms did not indicate that two animals were calling at the same time, as there were no overlapping calls (data not shown), we aimed to control for these possibly confounding effects by repeating the experiment, using anaesthetized ‘intruders’. We expected that resident female and male mice produce a similar number of USVs in response to vivid and anaesthetized intruders, as males were previously shown to respond to urinary samples of females with calling behavior [14].

**Table 1:** Percentage of call types usage (Mean +− SEM).

	Male/vivid Male	Male/vivid Fem	Fem/vivid Fem	P-values
CT1 (%)	29.6±6.5	**19.1±1.7**	**33.4±4.3**	**0.018**
CT2 (%)	52.9±7.4	50±3.2	42.8±4	0.578
CT3 (%)	17.4±8	30.6±4.3	23.9±5.5	0.578

P-values showed the results of the LMM with resident-intruder design as fixed factor and subject as random factor. Post hoc comparison was done with the least significant difference method (LSD). P-values of different call types were corrected for multiple testing (Simes correction). Significant differences are marked bold.

**Table 2:** Acoustic differences in relation to resident-intruder design (Mean +− SEM).

	Male/vivid Male	Male/vivid Fem	Fem/vivid Fem	P-values
Duration [ms]	40.9±7.7	61.3±6.4	54.5±5.9	0.557
Amplitude gap [%]	5.5±0.6	7.1±0.7	7.2±1	0.756
PF start [kHz]	75.2±1.1	73.5±1.2	75.4±0.8	0.557
PF max [kHz]	87.5±3	85.2±1.2	86.8±1	0.372
PF max loc [1/duration) * loc]	0.37±0.05	0.34±0.01	0.42±0.03	0.36
PF jump [kHz]	18.6±4.7	19.4±1.9	13.1±1.2	0.372
PF jump loc [1/duration) * loc]	0.39±0.04	0.37±0.01	0.43±0.03	0.372
Slope of trend	−0.14±0.07	−0.07±0.02	0.04±0.03	0.557

P-values showed the results of the LMM with resident-intruder design as fixed factor and subject as random factor. P-values were corrected for multiple testing (Simes correction).

### Experiment 2

The responses to anaesthetized intruders followed a similar pattern as the responses to vivid intruders. In response to female intruders, female mice called significantly more than males (t-test, t = −3.5, N = 37, P = 0.001), and overall produced the highest number of calls. In addition, males called more in response to anaesthetized female than male intruders (paired t-test, t = −2.5, N = 19, P = 0.022; Figure 1C). Resident males tended to reveal a shorter latency in response to intruding females than males (paired t-test: t = 2.2, N = 9, P = 0.064; Figure 1D). In terms of the call structure given by male residents to anaesthetized male or female intruders and female residents to anaesthetized female intruders we did not find any significant differences in call type usage (Table 3) or in acoustic variables (Table 4).

**Table 3:** Percentage of call types usage (Mean +− SEM).

	Male/anaesth. Male	Male/anaesth. Fem	Fem/anaesth. Fem	P-values	P-values vivid-anaesth.
CT1 (%)	35.6±5.44	37±4.4	47±4.6	0.681	**<0.001**
CT2 (%)	56.5±5.1	49.8±4.7	41.7±3	0.681	0.166
CT3 (%)	7.9±2.3	12.9±2.6	11.4±2.9	0.681	**0.003**

P-values showed the results of the LMM with resident-intruder design as fixed factor and subject as random factor. P-values were corrected for multiple testing (Simes correction). Significant differences are marked bold.

**Table 4:** Acoustic differences in relation to resident-intruder design (Mean +− SEM).

Acoustic parameters	Male/anaesth. Male	Male/anaesth. Fem	Fem/anaesth. Fem	P-values	P-values vivid-anaesth.
Duration [ms]	30.7±2.4	34.8±3.3	34.9±4.4	0.741	**<0.001**
Amplitude gap [%]	5.2±0.6	5.7±0.9	7.4±0.8	0.741	0.337
PF start [kHz]	75.4±1.4	73.8±0.8	73±1.2	0.741	0.978
PF max [kHz]	90.6±3.2	90.2±2.3	89.3±1.1	0.741	**0.004**
PF max loc [1/duration) * loc]	0.42±0.05	0.48±0.06	0.62±0.03	0.741	**<0.001**
PF jump [kHz]	17±2.1	15.3±1.2	10.8±1	0.741	**0.03**
PF jump loc [1/duration) * loc]	0.37±0.03	0.4±0.03	0.43±0.02	0.741	0.978
Slope of trend	0.04±0.07	0.17±0.11	0.27±0.05	0.741	**<0.001**

P-values showed the results of the LMM with resident-intruder design as fixed factor and subject as random factor. P-values were corrected for multiple testing (Simes correction).

We found no significant differences in the number of calls given to vivid or anaesthetized intruders (LMM: F_1,66_ = 0.83, p = 0.366), and marginally significant differences in the latency to start calling (LMM: F_1,25_ = 3.8, p = 0.063). In contrast, we found significant differences in the structure of calls given in response to vivid compared to anaesthetized intruders (Table 3 last column, Table 4 last column). In response to vivid intruders, male and female residents used fewer short calls with ascending frequency (CT1), and instead more long calls with frequency jumps (CT3; Table 1, 3). Accordingly, we found significantly longer calls with higher frequency jumps in response to vivid intruders. In addition, calls given in response to vivid intruders showed a significantly lower maximum peak frequency, an earlier location of the maximum, and the slope was generally more negative (Table 2, 4).

**Figure 3:**
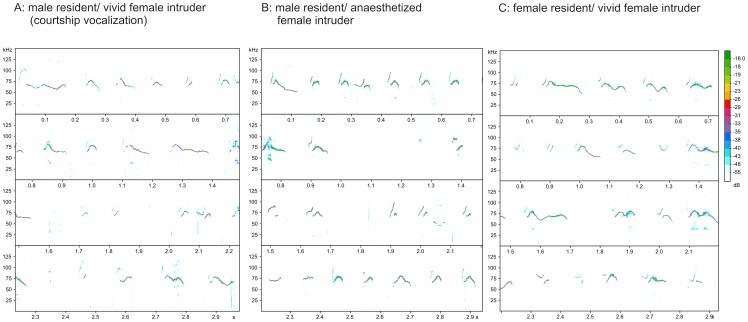
Examples of call sequences. Examples demonstrating the similar complexity of mouse call bouts. A: male resident/ vivid female intruder (courtship vocalization), B: male resident/ anaesthetized female intruder, C: female resident/ vivid female intruder.

Despite the fact that we found significant differences in a number of acoustic parameters, we did not found condition specific call types. A discriminant function analysis with all eight acoustic parameters used in the analysis had difficulties to assign the calls reliably to the experimental conditions (correct assignment: 50.7%; cross validated: 50.1%, chance level: 33.3%). For male calls given to vivid female intruder (courtship vocalization) we found a correct assignment of 43.9%. The assignment of female calls given to vivid female intruder did not get a better result (correct assignment: 49.8%). Only male calls given to anaesthetized female intruder reached a marginal better result (60.3%). Figure 3 illustrates the overall similarity of calls given by males and females, and in response to vivid and anaesthetized intruders.

The results indicate that the use of anaesthetized intruders is sufficient to elicit calling behavior from both females and males. The subtle differences in the acoustic structure indicates that subjects perceived the situations differently, perhaps as less arousing, as the calls given in response to anaesthetized intruders were shorter and exhibited fewer frequency jumps. These results support earlier findings that quantity and quality of emitted USV can be a useful marker to distinguish different contextual and motivational states [1], [19], [20], [21].

**Table 5:** Description of call parameter used in the analysis.

Acoustic parameters	Description
Duration [ms]	Time between onset and offset of call
Amplitude gap [ms]*	Duration of breaks in amplitude within call
PF start [Hz]	Start frequency of peak frequency
PF max [Hz]	Maximum peak frequency
PF max loc [(1/duration) * loc]	Location of PF max in relation to total call duration
PF jump [Hz]	Maximum difference of peak frequency between successive bins
PF jump loc [(1/duration) * loc]	Location of maximum PF jump in relation to total call duration
Slope of trend	Factor of linear trend of peak frequency

* In comparison between resident-intruder designs measured as percentage of call duration.

### General discussion

The findings that females responded generally most strongly, and that there were no significant acoustic differences in the acoustic structure of female and male calls questions the assumption that songs should primarily be considered as “male courtship signals” [13], [16]. Studies of wild animals also reported that males and females have a similar vocal behavior; California mice (*Peromyscus californicus*) produced USVs in the same contexts [22]. However, these mice have a different social system and a very simple structure in their USVs in comparison to the complex USVs of the mouse strain we used in this study. Although we refute the assumption that all calls are “courtship songs”, we cannot exclude the possibility that mouse songs have a variety of different and partly overlapping functions. This is not unusual. Bird song, for instance, typically functions to mark a male's territory and to attract females [23], while male baboon loud calls are used as displays of fighting ability as well as alarm calls [24]. The idea that mouse songs may serve different functions is compatible with the findings that the hormonal status of males has a crucial influence on whether the male starts to call [25] in response to females and that such call sequences can be elicited by female sex pheromones alone [26], [27]. It appears unlikely though that this is the sole function of these calls. This view is also supported by playback experiments [15], [28] showing strong habituation of female towards male songs, which is an untypical response for advertisement calls [29].

The view that female and male USVs given during these social encounters function as territorial signals is bolstered by two observations. Firstly, in studies with anesthetized residents, intruders produced only few or no vocalizations [30], [31], while several studies demonstrated that during social encounter the USVs are predominantly given by the resident animal [30]–[33]. Secondly, females in our study were only motivated to call when they were placed alone for more than one day in their “home cage”. During our pilot studies, we found that females which were only briefly moved to a new cage typically remained silent in response to “intruders”. This may also be the reason why we were unable to replicate the finding by Maggio and colleagues [18], who reported that female residents fail to call in response to anaesthetized male intruders.

The use of anaesthetized intruders appears to be a viable alternative to the use of vivid intruders. Because mice mostly do not show any overt signs when they produce calls, it is difficult to ascertain the identity of the caller. In animals, which communicate at larger distances it is possible to recognize the emitter by using the phase differences of the signal [34]. However, resident-intruder encounters happen at short distances and most USVs are given in close body contact. Therefore, it is not possible to use the phase differences to recognize the caller. With the use of anaesthetized intruders, the calls can be unambiguously assigned to the subject. The generally similar response of resident to vivid and anaesthetized intruders makes this approach a valuable tool for studies in which it is necessary to ascertain the identity of the caller, and is thus encouraged, although it should be clear that motivational or arousal changes do occur, which affect details of the acoustic structure of calls.

In sum, our results revealed more similarities than differences in the acoustic structure of male and female mouse call sequences, suggesting that these calls are generally social signals used by resident animals in response to intruders, and perhaps to regulate dominance relationships. At the same time, males may use these calls to attract females [15], although this is clearly not their sole function.

## Methods

### Animals, housing condition and ethic statement

C57BL/6NCrl female and male mice (Charles River, Sulzfeld, Germany), 8 weeks old upon arrival, were housed in groups of five in standard (Type II long) plastic cages, with food and water ad libitum. The temperature in the colony room was maintained at 20–22°C, the light-dark cycle at 12 h (light on at 08∶00). After 7 days of acclimatizing to the new environment, mice of a given cage were assigned randomly to either the group of residents or intruders. We used the same 20 males for all four male resident encounters; one male however became sick and did not participate in the experiment with the anaesthetized intruder. There was a seven-day interval between the different conditions, and the order of conditions was balanced. We used 20 resident females; 17 in the encounter with an anaesthetized female intruder, five of which also took part in the encounter with the vivid female intruder. The three remaining females participated in the encounter with the vivid female intruder only. Anaesthetized intruders were used three to four times in succession; vivid intruders were used three to four times but were exchanged after every single encounter. Resident mice were separated and housed individually in Type II standard cages for 7 days. The housing of intruder mice was not changed. All experiments were performed with permission of the local authorities (Bezirksregierung Braunschweig) in accordance with the German Animal Protection Law.

### Experimental procedure

We used the following conditions: females were confronted with vivid and anaesthetized female intruders, while males were confronted with both vivid and anaesthetized male and female intruders. Because males were expected to call more than females, we omitted the vivid male intruder condition. Because there was no condition to compare to, we also refrained from incorporating the experiment with anaesthetized males.

At the day of recording, intruder mice (males and females) were divided into two groups: “Vivid intruders” and “anaesthetized intruders”. Intruder mice were anaesthetized with an intraperitoneal (i.p.) injection of 0.25% tribromoethanol (Sigma-Aldrich, Munich, Germany) in the dose 0.125 mg/g of body weight. Vivid intruders were not treated. The estrus cycle phase of the female mice used in our current experiments was not investigated. Ten minutes before initiation of recording both residents and vivid intruder mice were woken up and kept awake by gentle shaking of the home cage in order to achieve a similar arousing level.

For the recording, resident mice (males and females) were first habituated to the room: Mice in their own home cage were placed on the desk in the recording room for 60 seconds. Subsequently, an unfamiliar intruder mouse was placed into the home cage of resident, and the vocalization behavior was recorded for 3 min using AVISOFT RECORDER 4.1. We recorded ultrasonic vocalizations of male and female mice at a sampling frequency of 300 kHz. The microphone (UltraSoundGate CM16) was connected to a preamplifier (UltraSoundGate 116), which was connected to a computer (all sound recording hardware and software was from Avisoft Bioacoustics, Berlin, Germany).

### Acoustic analysis

We counted the number of calls per recording session with AVISOFT Recorder 4.1. To separate USVs from other sound of the recording we used the whistles detection algorithm of AVISOFT Recorder with following selection criteria: Possible changes per step  = 4 (4687 Hz), minimal continuity  = 8 ms, possible frequency range  = 35 to 150 kHz. These criteria were compared with former analysis of male mouse vocalizations [2], [3], [5]. In addition, we visually inspected 5 percent of the recordings to ensure that the automated sampling routine selected only mouse USV and no other sounds such as toe clicking or sniffing. The AVISOFT recorder software stores the selected sounds in separate wave files, and, in addition logs the time of call onset.

From the stored calls, we calculated spectrograms (frequency range: 150 kHz, frequency resolution: 293 Hz, time resolution: 0.21 ms). We submitted the resulting spectrograms to the custom software program LMA 2011 to extract a set of characteristic acoustic parameters. As mice typically concentrate the energy of their USV in one small frequency band, so-called ‘pure tone-like sounds’ or ‘whistles’, we focused on peak frequency of USV, i.e. the loudest frequency of a respective time frame. Mice produce often soft sounds and just small head movements can lead to strong amplitude fluctuations in USVs. Therefore, we visually controlled the estimation of acoustic parameters and excluded incorrect estimated calls from the analysis. For each call we determined the duration of a call and the duration of amplitude gaps within a call. We defined the start of a call when the sound energy of a time segment is above 10% of the mean maximum amplitude of this call. An amplitude gap is defined if the sound energy of a certain time segment goes below 10%. To determine the end of a call we used a threshold of 15% of the mean maximum amplitude of a call. Furthermore, we determined start, maximum peak frequency, as well as the greatest difference in peak frequency between two consecutive 0.21 ms bins (so-called frequency jumps). In addition, we calculated the location of the maximum frequency and the location of peak frequency jump within the call. To describe the call modulation we calculated the slope of a linear trend through the peak frequencies of consecutive 0.21 ms bins. A detailed description of the estimated acoustic parameters is given in table 5. In total we analyzed 5278 calls, ranging from 4 (male vivid male encounters) to 96 calls per encounter, with a mean of 71.3.

### Statistics

Despite the jumps in peak frequency, all other acoustic parameters showed more or less continuous distributions. Therefore, we used a two-step cluster analysis (CA, PASW 18) to try to establish vocal categories. We calculated the clusters with all calls of all six encounters. We used the log-likelihood distance measure and Schwarzsches Bayes criteria (BIC) to find the best number of cluster. We used the eight acoustic parameters described above to calculate the CA. A higher number of parameters would have no advantages, because highly correlating acoustic parameters make it difficult to find appropriate cluster centers. In addition, they shift the result in the direction of correlating parameters. We conducted a discriminant function analysis (DFA, PASW 18) to confirm the cluster solution and estimated how the eight acoustic parameters contributed to the classification. We used the same DFA to assign the calls to the experimental conditions. Because we had the same resident males in the experiments we used a linear mixed model with experimental conditions as fixed factor and subject as random factor (PASW 18). We conducted separate tests for the three different call types (table 1 and 3) and for the eight different acoustic parameters (table 2 and 4). We corrected all p-values for multiple testing using Simes correction. Because of the ambivalent experience with the reliability of p-values in linear mixed model tests, we run a T-test in cases in which it was not necessary to use a mixed model.
